# Discovering and Differentiating New and Emerging Clonal Populations of *Chlamydia trachomatis* with a Novel Shotgun Cell Culture Harvest Assay

**DOI:** 10.3201/eid1403.071071

**Published:** 2008-03

**Authors:** Naraporn Somboonna, Sally Mead, Jessica Liu, Deborah Dean

**Affiliations:** * Children’s Hospital Oakland Research Institute, Oakland, California, USA; † University of California, Berkeley, California, USA; ‡ University of California School of Medicine, San Francisco, California, USA

**Keywords:** Chlamydia trachomatis, clone cells, plaque assay, omp1, 16S ribosomal RNA, plasmid, research

## Abstract

*Chlamydia trachomatis* is the leading cause of preventable blindness and bacterial sexually transmitted diseases worldwide. Plaque assays have been used to clonally segregate laboratory-adapted *C. trachomatis* strains from mixed infections, but no assays have been reported to segregate clones from recent clinical samples. We developed a novel shotgun cell culture harvest assay for this purpose because we found that recent clinical samples do not form plaques. Clones were strain-typed by using outer membrane protein A and 16S rRNA sequences. Surprisingly, ocular trachoma reference strain A/SA-1 contained clones of *Chlamydophila abortus*. *C. abortus* primarily infects ruminants and pigs and has never been identified in populations where trachoma is endemic. Three clonal variants of reference strain Ba/Apache-2 were also identified. Our findings reflect the importance of clonal isolation in identifying constituents of mixed infections containing new or emerging strains and of viable clones for research to more fully understand the dynamics of in vivo strain-mixing, evolution, and disease pathogenesis.

## Abstract 2

This assay, coupled with ompA and 16S rRNA sequencing, characterized clonal populations of *C. trachomatis*.

*Chlamydia trachomatis* is a ubiquitous human pathogen that is responsible for the most prevalent bacterial sexually transmitted diseases (STDs) worldwide (*1*). As an obligate intracellular bacterium, it has a distinctive biphasic developmental cycle (*2*). The cycle begins when metabolically inactive elementary bodies (EBs) infect the host cell and reside in a vacuole termed an inclusion body. EBs differentiate into noninfectious, metabolically active reticulate bodies that multiply by binary fission and redifferentiate into EBs after ≈30–48 hours and then are released from the cell by lysis or exocytosis to initiate a new round of infection (*2*).

The organism comprises 2 biovars, trachoma and lymphogranuloma venereum (LGV) (*3*). These biovars comprise 19 serologic variants (serovars), which are identified by monoclonal antibodies that react to epitopes on the major outer membrane protein (MOMP) (*4*). Variants of *ompA*, the gene that encodes MOMP, differentiate genotypes of these serovars (*5**–**7*). Phylogenetic analyses and statistical modeling have enhanced *ompA* genotyping. For example, serovar B is restricted to the ocular mucosa while Ba is found in the eye and urogenital tract (*8*). The LGV biovar (L_1_, L_2_, L_2_′, L_2_a, L_2_b, L_3_) causes invasive STDs (*9**,**10*). The trachoma biovar (A, B, Ba, C, D, Da, E, F, G, H, I, Ia, J, Ja, K) is responsible for ocular disease, termed trachoma, and for STDs globally. The former is caused by serovars A to C and Ba and the latter by D through K, Da, Ia, Ja, and rarely Ba and C (*4*,*5**,**11*).

Approximately 8%–57% of clinical STD samples mixed infections (*5**–**7**,**9**,**12**,**13*). Thus, an inherent problem with strain typing is detecting mixed infections. These infections can be identified by using PCR primers that are specific for each strain followed by sequencing (*5*), by cloning PCR products and sequencing >10 clones (*11**,**13*), or by reverse dot-blot hybridization of PCR amplicons to serovar-specific probes (*14*). However, none of these techniques can detect new genetic strains that fail to either anneal with the current selection of primers or hybridize with the available probes. The end product is also a nonviable DNA sequence.

Increasingly, isolates representing single clones are needed for in vitro and in vivo research, including genomic, murine, and translational studies, to advance our understanding of chlamydial pathogenesis. Although a few studies have described methods for segregating clones of laboratory-adapted *C. trachomatis* clinical and reference strains (*12**,**15**,**16*), none has clonally purified all 19 *C. trachomatis* reference strains nor determined optimal methods to clonally segregate clinically mixed samples. Consequently, we modified the plaque-forming assay of Matsumoto et al. (*16*) to segegrate clones from reference strains and developed a novel cell culture shotgun harvest assay to segregate viable clones from recent clinical samples because typical plaques do not form for most of these samples.

Our culture techniques coupled with outer membrane protein A (*ompA* ) and 16S rRNA sequencing identified the constituents of mixed infections that represented new and emerging *Chlamydiaceae* strains and clonal variants in human disease. These results stress the importance of clonal isolation for these types of discoveries. Clonal isolates will also be essential for chlamydial research to ensure reproducibility of experiments among laboratories and to understand the dynamics of in vivo strain-mixing, evolution, and disease pathogenesis.

## Materials and Methods

### *C. trachomatis* Reference and Clinical Strains

We studied 19 *C. trachomatis* reference strains (A/SA-1, B/TW-5, Ba/Apache-2, C/TW-3, D/UW-3, Da/TW-448, E/Bour, F/IC-Cal3, G/UW-57, H/UW-4, I/UW-12, Ia/IU-4168, J/UW-36, Ja/UW-92, K/UW-31, L1/440, L2/434, L2a/TW-396, and L3/404) and 5 clinical strains, representing *ompA* genotypes G, F, H, Ja, and K (Table 1). Reference strains were the original isolates. A/Har-13; *Chlamydophila caviae*, strain GPIC; *Chlamydia muridanum*, strain Nigg; *Chlamydophila abortus*, strain S26/3; and another seed stock of A/SA-1 were included for PCR amplification analyses (see Preparation of Genomic DNA and Sequencing of *ompA* and 16S rRNA for Each Clone). Clinical strains were isolated from acute (Ja and K strains; no prior history of chlamydial STD) and persistent cervical strains (F, G, and H; same-ompA genotypes occurring in the same woman over several years despite antimicrobial drug therapy). Clinical samples were identified by a unique identification number with no link to patient names.

**Table 1:** Results of the modified plaque assay for *Chlamydia* reference strains and shotgun cell culture harvest technique for clinical strains representing acute and persistent infections*

Strain	Days p.i. to plaque formation or harvest	No. plaques or shotgun harvested areas	*ompA* genotype (no.)	Location of nucleotide substitutions in *ompA* (amino acid substitution location)	16S rRNA (no.)
Reference D/UW-3 and E/Bour 1:1†	9	25	D (13) E (9) D/E (3)	– – –	*Chlamydia trachomatis* D (13) *C. trachomatis* E (9) *C. trachomatis *D/E (3)
Reference D/UW-3 and E/Bour 3:‡	9	9	D (9) E (0)	– –	*C. trachomatis* D (9) *C. trachomatis* E (0)
D/UW-3/E/Bour mixed infection§	9	11	D (7) E (4)		*C. trachomatis* D (7) *C. trachomatis* E (4)
Acute clinical Ja	8	11	Ja (11)	–	*C. trachomatis *Ja (11)
Acute clinical K	10	11	K (11)	–	*C. trachomatis* K (11)
Persistent H	7	5	H (5)	–	*C. trachomatis* H (5)
Persistent G	14	7	G (7)	–	*C. trachomatis* G (7)
Persistent F	10	5	F (5)	–	*C. trachomatis* F (5)
Persistent clinical F and G strains 1:1†	10	13	F (1) G (12)	– –	*C. trachomatis* F (1) *C. trachomatis* G (12)
A/SA-1	10 10¶	18 21	A (14) *Chlamydophila abortus* (4) A/*C. abortus* (21)	– –	*C. trachomatis* A (14) *C. abortus* (4) *C. trachomatis *A/*C. abortus* (21)
B/TW-5	9	15	B (15)	–	*C. trachomatis* B (15)
Ba/Apache-2	8	14	Ba (9) Ba_1_ (1) Ba_2_ (1) Ba_3_ (1)	– **C662T (P221L)** **C662T (P221L) G673A (E225K)** **C662T (P221L)** A717C (K239N)	*C. trachomatis *Ba (14)
C/TW-3	12	13	C (13)	–	*C. trachomatis* C (13)
D/UW-3	9	11	D (11)	–	*C. trachomatis* D (11)
Da/TW-448	10	12	Da (12)	–	*C. trachomatis *Da (12)
E/Bour	9	9	E (9)	–	*C. trachomatis* E (9)
F/IC-Cal3	9	13	F (10) F-III (3)	– G269A (G90**E**)#	*C. trachomatis* F (13)
G/UW57/Cx	7	10	G (10)	–	*C. trachomatis* G (10)
H/UW-4	12	12	H (10)	–	*C. trachomatis* H(10)
I/UW-12	11	14	I (14)	–	*C. trachomatis* I (14)
Ia/IU-4168	12	11	Ia (12)	–	*C. trachomatis *Ia (12)
J/UW-36	11	15	J (15)	–	*C. trachomatis* J (15)
Ja/UW-92	12	12	Ja (12)	–	*C. trachomatis *Ja (12)
K/UW-31	11	13	K (13)	–	*C. trachomatis* K (13)
L_1_/440	9	11	L_1_ (11)	–	*C. trachomatis *L_1_ (11)
L_2_/434	9	11	L_2_ (8) L_2_’ (3)	– C471G, G496A (A166T)¶	*C. trachomatis *L_2_ (10) *C. trachomatis *L_2_a (1)
L_2_a/TW-396	9	13	L_2_a (13)	–	*C. trachomatis *L_2_ (13)
L_3_/404	9	14	L_3_ (14)	–	*C. trachomatis *L_3_ (14)

*p.i.,.postinoculation; ompA, outer membrane protein A; –, no evidence for nucleotide substitution. †Represents an equal mixture of inclusion-forming units (IFUs) of each strain. ‡Represents a 3:1 mixture of IFUs of reference strains D/UW-3 and E/Bour. §Represents the plaques from the 1:1 mixture of D/UW-3 and E/Bour when both reference strains were found in 3 plaques. ¶Represents the plaques that were harvested from the wells with 10^–8^ dilution. #**Boldface** denotes nonconservative amino acid substitution (*17*).

### *C. trachomatis* Culture and Titration of Inclusion-Forming Units

Confluent monolayers of McCoy cells were inoculated with reference and clinical strains by centrifugation at 550 × *g* for 1 h at 35°C. Cultures were maintained at 37°C and 5% CO_2_ in chlamydial growth medium (CMGH), which contains minimal essential medium (MEM; Cellgro, Mannassas, VA, USA]; 10% fetal bovine serum (FBS; University of California, San Francisco [UCSF] Cell Culture Facility, San Francisco, CA, USA); 0.45% glucose solution (Cellgro); 20 mmol/L HEPES (UCSF Cell Culture Facility); 0.08% NaHCO_3_, 10 μg/mL gentamicin (MP Biomedicals, Solon, OH, USA); 25 µg/mL vancomycin (Acros Organics, Morris Plains, NJ, USA); 25 units/mL nystatin (MP Biomedicals), 375 µg/mL amphotericin B (Pharma-Tek, Huntington, NY, USA); 1 μg/mL cyclohexamide for 48 h and harvested as described (*18**,**19*). Inclusion-forming units (IFUs) were titrated after 30–48 h of growth, depending on the strain, by using chlamydial lipopolysaccharide (LPS)–specific monoclonal antibodies (LPS-MAbs; Virostat, Portland, ME, USA) (*2**,**18*).

### Plaque Assay for Reference Strains and Clinical Samples

We modified the plaque assay of Matsumoto et al. (*16*) by using low speed centrifugation at 550 × *g* and 6-well plates for infections, and 1-dram shell vials (Kimble Chase Inc., Vineland, NJ, USA) for propagation. To ensure detection of mixed infections, 1:3 and 1:1 ratios of IFUs for reference strains E/Bour and D/UW-3, and a 1:1 ratio for clinical strains F and G, were created for inoculation and harvest.

Reference and clinical strains were serially diluted in sucrose-phosphate-glutamine (SPG) (219 mmol/L sucrose; 3.82 mmol/L KH_2_PO_4_; 8.59 mmol/L Na_2_HPO_4_; 4.26 mmol/L glutamic acid; 10 μg/mL gentamicin; 100 μg/mL vancomycin; 25 U/mL nystatin in distilled water, pH 7.4). Each 6-well plate contained dilutions from 1.25 × 10^6^ IFUs in the 1st well to 1.25 × 10 IFUs on 60%–70% confluent McCoy cell monolayers. Two 6-well plates were prepared identically per strain except that the second plate contained a glass coverslip in each well. After centrifugation, the inocula were removed and replaced with CMGH plus 1 μg/mL cyclohexamide and maintained at 37°C in 5% CO_2_.

At 24 h postinfection (p.i.), culture medium was aspirated, and wells were overlaid with initial agarose (IAO: 0.5% SeaKem ME agarose [BMA, Rockland, ME, USA]) in phenol red–free MEM (BioWhittaker, Walkersville, MD, USA); 10% FBS; 1 μg/mL cyclohexamide. Two milliliters of CMGH without cyclohexamide were added to the solidified IAO. Medium was replaced every 4 days to optimize chlamydial growth.

Once small plaques formed by visual inspection at 7–12 days p.i., medium was removed, and final agarose overlay (FAO: 0.5% SeaKem ME agarose in phenol red–free MEM; 10% FBS; 1/100 volume of 3% neutral red [Sigma-Aldrich, St. Louis, MO, USA]) was dispensed onto IAO. CMGH, without cyclohexamide, was added, and the plates were incubated for 12–24 h.

At 48 h, duplicate plates with coverslips were fixed with methanol and stained with a fluorescein isothiocyanate (FITC)–conjugated *C. trachomatis* LPS-MAbs (*18*). Inclusions on each cover slip were counted to determine IFUs per milliliter per well and efficacy of infection given the calculated IFUs inoculated for each well.

**Figure 1:**
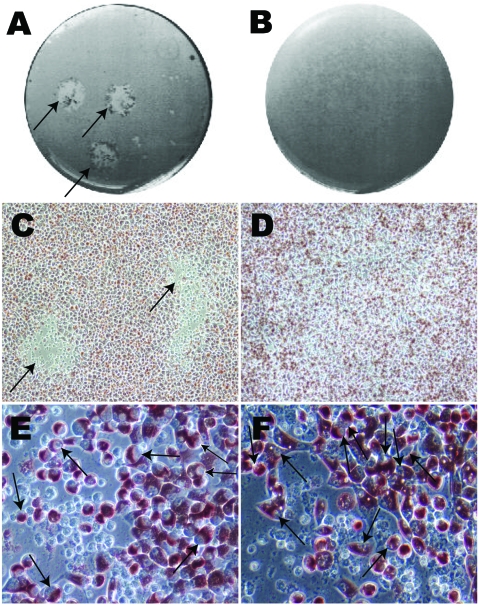
Photographs and optical microscopy views of the wells showing plaques formed by *Chlamydia trachomatis* F/IC-Cal3 (A, C, E) and no plaque formed by clinical F persistent strain (B, D, F). A) Single well showing 2 distinct plaques (indicated by arrows). B) Well showing no plaque morphology. C) and D) Optical microscopy image showing plaque areas with little or no neutral red staining (arrows) surrounded by viable cells stained red (magnification ×100). Higher magnification (×400) showed numerous cells that had been infected by reference strain F/IC-Cal3 (E) and the clinical persistent F strain (F).

Plaques were visualized as a central area of cellular debris surrounded by viable infected cells with red staining of cytoplasm at the cell periphery (Figure 1, **panels A and C**). Inclusion bodies and nonviable cells remained clear. Any plaque (≈1–2 mm) that was clearly isolated from another plaque, or appeared as a solitary plaque in a well, was selected. A blunt-ended transfer pipette was used to punch a hole ≈1–2 mm in diameter through the gels over the plaque. The contents were placed into a microcentrifuge tube containing CMGH, sonicated and added to shell vials containing McCoy monolayers for propagation. Centrifugation of shell vials at 2,400 × *g* for 1 h at 35°C was required to successfully grow each clonally segregated strain. Strains were propagated and purified using gradient ultracentrifugation as previously described (*2**,**18**–**20*). The pellet was resuspended in SPG, and stored at –80°C.

### Shotgun Harvest, Isolation, and Propagation of Single Clonal Populations for Clinical Strains

**Figure 2:**
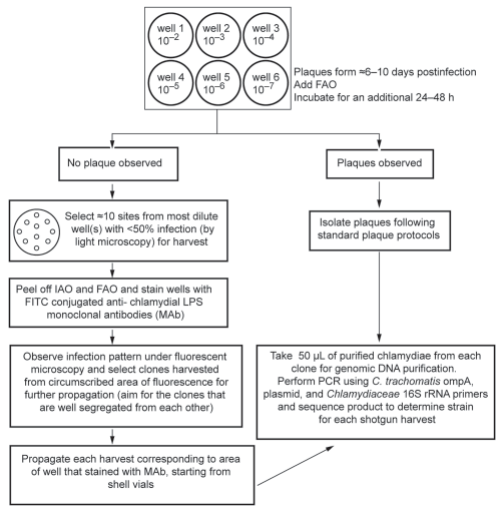
Diagram of the cell culture and shotgun harvest assay for *Chlamydia trachomatis* clinical strains propagated in McCoy cells. Serial dilutions of each clinical sample were used for inoculating wells 1 to 6. IAO, initial agarose overlay; FAO, final agarose overlay.

Because no visible plaques formed for the clinical strains, except for clinical H, the plates were inspected under 100× and 400× light microscopy. Wells were selected for our shotgun harvest as shown in the diagram (Figure 2). Ten spots per well were numbered where the infections were observed under microscopy. Each spot was harvested (≈2–3 wells × 10 spots per well = 20–30 harvests) using a sterile, blunt-ended transfer pipette.

IAO and FAO were carefully removed, and the wells were stained using FITC-conjugated *C. trachomatis* LPS-MAb (Virostat). Only harvested areas that corresponded to a confined group of infected cells with a clear margin from uninfected cells were selected, sonicated, inoculated, propagated in shell vials and flasks purified and stored as above. The original clinical samples were also independently propagated in shell vials as described previously (*2**,**18**–**20*) for comparison with growth in the plaque assay.

### Preparation of Genomic DNA and Sequencing of *ompA* and 16S rRNA for Each Clone

Purified culture was used for genomic DNA extraction according to High Pure Template Preparation Kit package insert (Roche Diagnostics**,** Indianapolis, IN, USA). PCR was performed and reagents, thermocycling profile, and sequencing were used according to previously described protocols (*21*). Table 2 (*22*) shows the primers used for PCR and sequencing to identify the strain-type of each clone. Multiple sequences were aligned by using MegAlign software (DNASTAR, Madison, WI, USA) and compared with public sequences (*21**,**23*). A variant was defined as having >1 nucleotide difference(s) from the sequence of the reference strain for either *ompA* or 16S rRNA genes.

**Table 2:** PCR and sequencing primers used for determining strain types of clonal isolates from reference strains and clinical samples

Primer	Sequence (5′ → 3′)	Location	Ref
CTompA-F	GTCCCGCCAGAAAAAGATAG	–60 to –41	This study
CTompA-seqF	ATAGCGAGCACAAAGAGAGC	–44 to –25	This study
VB3	CATCGTAGTCAATAGAGGCAT	817 to 797	(*22*)
MVF3	TGTAAAACGACGGCCAGTGCCCGTGCAGCTTT	561 to 611	(*22*)
CTompA-B	ACGGATAGTGTTATTAACAAAGAT	1261 to 1225	This study
CTompA-seqB	GTAAAACGACGGCCAGT	562 to 596	This study
C16SrRNA-F	CAGTCGAGAATCTTTCGCAAT	359 to 380	This study
C16SrRNA-seqF	AAGGCTCTAGGGTTGTAAAGCACTTT	419 to 444	This study
C16SrRNA-B	TACTGGCCATTGTAGCACGTGTGT	1230 to 1253	This study
Plasmid-PF5	AGACTTGGTCATAATGGACTT	1022 to 1002	This study
Plasmid-seqPF5	AGACTTGGTCATAATGGACTT	1022 to 1002	This study
Plasmid-PB5	TTGTCTCGGATTTTAAAAAATGT	588 to 566	This study
FCabortus	GGTATGTTTAGGCATCTAAAA	172 to 192	This study
RCabortus2	GGCCATTGTAGCACGTGTGTA	1248 to 1228	This study

### Phylogenetic Construction of *ompA* Nucleotide and Amino Acid Sequence Alignments

Nucleotide and amino acid alignments and phylogenetic analyses of the 19 reference strains and clonal variants were performed by using MEGA 3.1 (Center for Evolutionary Functional Genomics, Tempe, AZ, USA) as described (*21**,**23*). Briefly, neighbor-joining trees were calculated using the Kimura 2-parameter model that assumes that nucleotide frequencies and rates of substitution do not vary among sites. For amino acids, neighbor-joining trees were calculated using the gamma distance model that considers the dissimilarity of substitution rates among sites. We used bootstrap analysis (1,000 replicates) to determine confidence intervals for each branch.

## Results

### Plaque Formation by Reference *C. trachomatis* Strains

Figure 1, **panel A**, shows typical plaque formation for F/IC-Cal3. Higher inocula resulted in plaques that fused and, therefore, were not suitable for harvest. These findings are similar to those of others who have used plaque- or focus-forming assays for clonal segregation of laboratory-adapted chlamydial strains (*16**,**24*).

Table 1 shows the day p.i. that plaques were visualized and the number of isolated clones and nucleotide polymorphisms with respect to reference strain sequences. All reference strains formed mature plaques ≈1–2 mm in diameter. Experimentally mixed infections of D/UW-3 and E/Bour resulted in 13 D, 9 E and 3 D/E clones, and 9 D and 0 E clones. The 3 D/E clones were identified as mixed based on electropherograms where 2 peaks were observed in a single nucleotide position that corresponded to D and E sequences for ≈20 nucleotide positions. These 3 mixed infections were further plaque-purified as above and yielded single clonal populations of D or E, which validated our plaque assay for isolating clonal populations.

### Detection of Clonal Populations of *C. trachomatis* Clinical Strains by Shotgun Harvest

The clinical strains Ja, K, F, and G showed no plaques, while persistent strain K showed signs of <0.5-mm plaques at 10 days p.i., and strain H showed typical plaques at 7 days p.i. A longer growth period up to 20 days p.i. did not result in distinct plaque formation for Ja, K, F, or G. Figure 1, **panels B and D**, show a well with an inoculum of 1.25 × 10^3^ at day 10 p.i. where no plaques were visualized.

**Figure 3:**
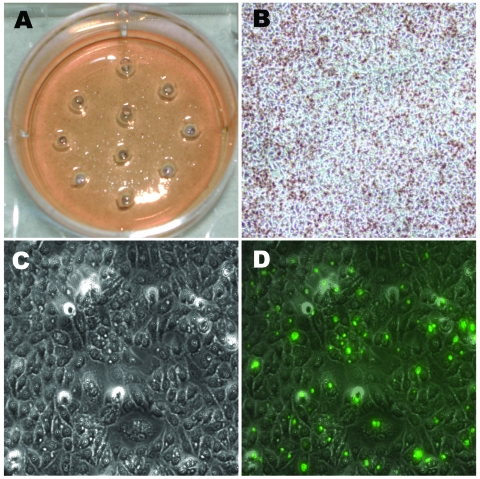
Photographs of light and fluorescent microscopy showing the shotgun cell culture harvest method for isolating clonal populations of clinically persistant strains. A) 1 well in a 6-well plate after harvesting 10 random areas using sterile pipettes; no plaques could be visualized in any well unlike the situation for the reference strains in Figure 2. B) The same well before harvesting infected areas (magnification ×100). C) Light microscopy view of an area with infected cells containing small inclusion bodies after agarose overlays had been removed (magnification ×400). D) Fluorescent microscopy view of the same field as in C (magnification ×400); infected cells were stained with *Chlamydia trachomatis–*specific lipopolysaccharide antibodies. Arrows denote small fluorescing inclusion bodies within the cell cytoplasm.

Approximately 20–30 regions of 2 mm each (Figure 3, **panel A**) from 2–3 wells per isolate were harvested for each of the clinical strains after light microscopic examination (Figure 3, panels B and C). On the basis of fluorescent microscopic examination of the wells after removal of the agarose layers and staining with FITC-conjugated LPS-MAb to detect segregated areas of chlamydial infection (Figure 3, **panel D**), 11 clinical Ja, 11 clinical K, 5 clinical H, 5 clinical F, and 7 clinical G harvests were selected for propagation.

Notably, the size of the inclusion body was much smaller for clinical strains than for reference strains. Figure 1, **panel E** shows typical large inclusion bodies formed by reference F/IC-Cal3 compared with tiny and occasional medium-sized inclusion bodies at day 10 for persistent clinical strain F (Figure 1, **panel F**). Similar results were observed for persistent clinical strains G and H when compared with respective reference strains. In contrast, acute clinical strains Ja and K had inclusion bodies that were intermediate in size (data not shown). When original clinical samples were propagated in shell vials, inclusions remained small and the rate of growth was similar as for the plaque assay.

Mixed clinical G and F strains yielded 12 G clones (92.31%), 1 F clone (7.69%), and no mixed clones based on sequencing. Figure 3, **panel A**, represents 1 well after 10 random areas were harvested since no plaque was visible (Figure 3, **panel B**). Figure 3, **panel C**, represents a microscope photo where chlamydial inclusions are difficult to visualize due to their small size. Figure 3, **panel D**, is a fluorescent image of Figure 3, **panel C**, displaying small and medium-sized inclusion bodies.

### Sequence Analyses of *ompA* and 16S rRNA *C. trachomatis* Clonal Populations

A total of 30 chlamydial *ompA* genotypes were identified from the plaque assay and shotgun harvest based on sequence analyses using BLAST and MegAlign as we have described (*21**,**22*) (Table 1). Three reference strains showed mixed infections: Ba/Apache-2 with new Ba *ompA* genotypes, Ba_1_, Ba_2_, and Ba_3_ (1 clone each), F/IC-Cal3 with F-III (*5*), L_2_/434 with L_2_′ (*9*), and A/SA-1 with *C. abortus* S26/3 (*25*), which were clonally segregated into 14 A and 4 *C. abortus* clones (Table 1). All 4 *C. abortus* clones had the same sequences for *ompA* and 16S rRNA.

The *C. abortus–*specific primers (Table 2) were used to amplify another seed stock of A/SA-1, *C. abortus* (from our plaque assay), *C. trachomatis* strain A/Har-13, and *C. caviae* and *C. muridanum*. For the *C. abortus*–specific PCR amplification, only A/SA-1 and *C. abortus* samples were positive, while the rest were negative.

### Phylogenetic Analyses of *ompA* *C. trachomatis* Reference and Clonal Populations

**Figure 4:**
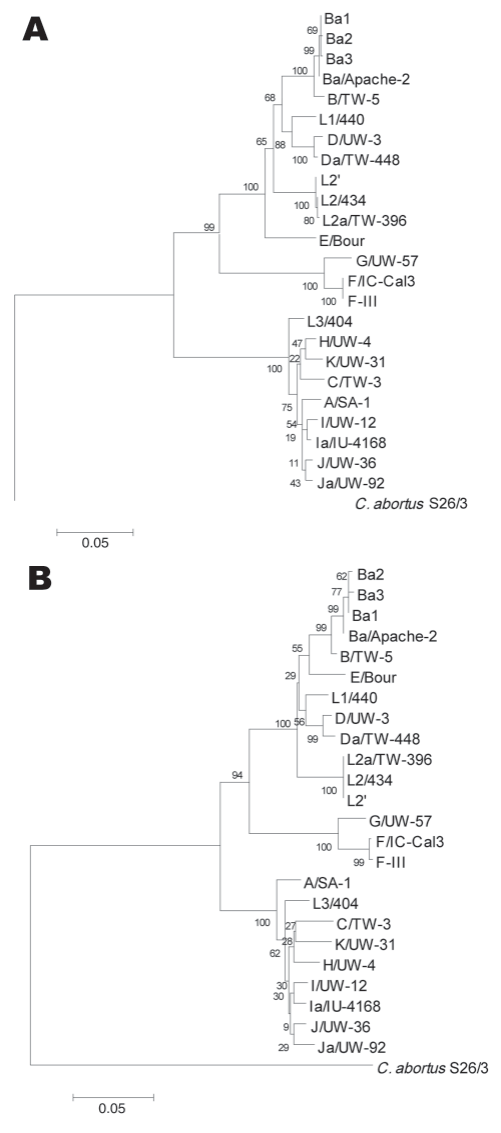
Neighbor-joining trees representing evolutionary relatedness of the 19 reference strains, *Chlamydophila abortus* and 5 clonal variants based on *ompA* nucleotide (A) and amino acid (B) sequence alignments. The trees were constructed from ClustalW 1.8 alignment (www.ebi.ac.uk/Tools/clustalw2/index.html), and the values at the nodes are the bootstrap confidence levels calculated from 1,000 bootstrap resamplings. See Materials and Methods for details.

Phylogenetics of *ompA* nucleotide and amino acid sequence alignments were performed to evaluate divergence of 5 clonal variants of Ba/Apache-2 and F/IC-Cal3. The trees showed the clustering of the 5 clonal variants with their respective parental strains (Figure 4, **panels A and B**).

## Discussion

Plaque- and focus-forming assays have been developed to isolate individual clonal populations of reference strains of *C. trachomatis* (*12**,**15**,**16*) and *Chlamydophila pneumoniae* (*24*). The first methods for *C. trachomatis* used L (*15*) and McCoy cells (*16*). More recently, flow cytometry has been used to segregate cells infected with *C. trachomatis*, *C. caviae,* and *Chlamydia suis* (*12*). However, these techniques have focused on laboratory-adapted clinical and reference strains and have not used nonpropagated or nonlaboratory-adapted clinical samples.

The novel shotgun harvest assay that we developed was successful in segregating clonal populations of *C. trachomatis* strains and variants that were devoid of plaque-forming characteristics. Consequently, our method is an important advance in reliably detecting and purifying clonal isolates from clinical samples. We also modified the plaque protocol of Matsumoto et al. (*16*), which allowed us to use lower concentrations of reference strains, ensuring widely separated or single plaques. Most important, our methods showed sample collections that contained mixtures of new and emerging strains and variants based on *ompA* and 16S rRNA sequences.

The most remarkable mixed infection was for reference strain A/SA-1 in which *C. abortus* was identified. *C. abortus* was not a likely contaminant because A/SA-1 was an original isolate**.** PCR of another seed stock of A/SA-1 was positive for *C. abortus*, and *C. abortus* had not previously been propagated in our laboratory. The original sample was obtained from the conjunctiva of a trachoma patient in Saudi Arabia in 1957. This finding was unexpected because *C. abortus* has not been described among trachoma-endemic populations. Although *C. abortus* may be responsible for zoonoses in pregnant women, it resides in a unique niche, the placenta, compared with *C. trachomatis* (*26*). Thus, an explanation for our findings is that *C. abortus* is now capable of crossing species or niche barriers. Indeed, we recently identified mixed conjunctival infections with *C. trachomatis*, *Chlamydophila psittaci,* and/or *C. pneumoniae* in 35% of infected persons residing in a trachoma-endemic region of Nepal (*27*). The findings were statistically unlikely to have occurred by chance. Additionally, infection with *C. pneumoniae* or *C. psittaci* was significantly associated with trachomatous inflammation, a precursor for scarring. With mounting evidence for widespread interstrain recombination among intracellular bacteria such as *Chlamydiaceae* (*8**,**10**,**21**–**23**,**28*), the A/SA1 coinfection with *C. abortus* along with those described above are likely the tip of the iceberg in terms of the prevalence of mixed *Chlamydiaceae* infections and the possibility for recombination that may result in diverged tissue tropism (*21**,**23*). We are currently examining samples from other trachoma-endemic populations for coinfection with *C. abortus* and other *Chlamydiaceae* species*.*

Reference strain Ba/Apache-2 also comprised clonal populations of 3 previously unrecognized *ompA* genotypes, Ba_1_, Ba_2_ and Ba_3_, that were distinct from publicly available Ba *ompA* sequences (*6**,**7**,**29*). The C662T mutation among our clones encoded a nonsynonymous P221L substitution in a constant region (CR) between variable segments (VSs) II and VSIII of MOMP, which contains 5 CRs and 4 VSs. This change from a proline, an imino amino acid with unique “kink,” to a nonpolar leucine on CRIII might disrupt the mid-portion ß-strand transmembrane of MOMP (*30**–**32*). Furthermore, the E225K in Ba_2_ occurs in VSIII where the subspecies-specific epitope for LGV and A–K strains (*32*) is located, likely changing polarity of the epitope from a negative to a positive charge. These mutations, then, may lead to adaptive structural and/or functional changes for MOMP.

The presence of mutations in Ba_1_, Ba_2_, and Ba_3_ suggests that these have occurred under immune selection in vivo, because growing reference strains in vitro has not shown detectable mutations (*3**,**14*), although in theory this could occur. On the basis of phylogenetic reconstructions (Figure 4), the clonal variants likely represent natural diversity arising from the respective parental strain. Also, the ability of Ba strains to either mutate specific protein regions or recombine may facilitate their invasion of other mucosal sites. Urogenital Ba infections do occur, and we have previously described a Ba/D recombinant that was isolated from the genital tract (*8*).

Notably, most of the *ompA* mutations were located within CRs and encoded for nonsynonymous substitutions, the majority of which encoded for nonconservative amino acids with altered properties. For instance, *ompA* genotype F-III contains a nonconservative G90E substitution. G90E is located in CRII next to VSI, which may decrease membrane hydrophobicity and disrupt the >0.5 nonpolar or hydrophobic index requirement for the MOMP spanning region (*32*). In 2 separate studies, we identified F-III variants as statistically significantly associated with pelvic inflammatory disease (PID) (*5**,**33*). The F-III mutation may explain, in part, the association with PID. However, additional studies will be required to delineate these associations.

In our experimentally mixed infections, recovery of separate clones of D/UW-3 and E/Bour, and of clinical G and F validated each assay (Table 1). The greater number of clones for D/UW-3 (52%) than for E/Bour (36%), and for G (92.31%) than for F (7.69%) might indicate different growth rates and timelines for plaque formation and characteristics of each strain (*15**,**16*). It is also possible that 1 strain produces byproducts of growth that are inhibitory for coinfecting strains. Nevertheless, these data emphasize the importance of selecting multiple wells of low inocula for plaque or shotgun harvests to identify all strains that are present. Additionally, mixed infections may occur where some strains cause plaque formation and others do not, which stresses the importance of the shotgun harvest even when the morphologic features of plaque are present.

In the present study, we analyzed clones by sequencing *ompA*, the plasmid, and 16S rRNA to enhance strain categorization. The plasmid was evaluated because its absence has been reported to correlate with reduced or no plaque formation (*34*). However, all of our clones contained the plasmid, which is consistent with other studies (*35**–**37*). The lack of classic plaque formation for clinical isolates likely stems from their slow growth and lack of adaptation to conventional cell culture. This was borne out by their slow growth in shell vials and flasks, experiments which were performed separately from the plaque assay. Clinical strains may exit the cell without cellular disruption, facilitating subsequent rounds of infection and lack of plaque formation. Beatty recently showed,that EBs could be released without lysis and also be retained by host cells (*38*). However, our clinical H formed plaques similar in morphology to reference strains. The presence of a complete toxin gene, as in H/UW-4 and J/UW-36 (*39*), may have contributed to clinical H plaque formation. H/UW-4 has been shown to produce more cytotoxicity than D/UW-3, which contains a partial toxin gene, and *C. muridanum*, which contains a full-length gene (*40*). Although all 19 reference strains formed classic plaques morphology, some have no toxin (LGV strains) or a partial gene, which suggests that plaque formation reflects adaptation to culture that has occurred over decades instead of the effects of the toxin. Further experiments will be required to determine the genetic factors involved in plaque formation.
